# An observational study of system-level changes to improve the recording of very brief advice for smoking cessation in an inpatient mental health setting

**DOI:** 10.1186/s12889-020-08672-y

**Published:** 2020-04-25

**Authors:** Gilda Spaducci, Sol Richardson, Ann McNeill, Megan Pritchard, Jyoti Sanyal, Andy Healey, Mary Yates, Debbie Robson

**Affiliations:** 1 Addictions Department, Institute of Psychiatry, Psychology & Neuroscience, King’s College London, 4 Windsor Walk, Denmark Hill, London, SE5 8BB UK; 2 Centre for Tobacco and Alcohol Studies, Nottingham, UK; 3 NIHR Maudsley Biomedical Research Centre & King’s College London, De Crespigny Park, Camberwell, London, SE5 8AF UK; 4 King’s Improvement Science and King’s Health Economics, Health Service and Population Research Department, Institute of Psychiatry, Psychology & Neuroscience, King’s College London. David Goldberg Centre, De Crespigny Park, London, SE5 8AF UK; 5 South London and Maudsley NHS Foundation Trust, Bethlem Royal Hospital, Beckenham, Kent, UK

**Keywords:** Very brief advice, Smoking cessation, Mental health, Psychosis, Hospitalised

## Abstract

**Background:**

Smoking prevalence among people with psychosis remains high. Providing Very Brief Advice (VBA) comprising: i) ASK, identifying a patient’s smoking status ii) ADVISE, advising on the best way to stop and iii) ACT (OFFER), offering a referral to specialist smoking cessation support, increases quit attempts in the general population. We assessed whether system-level changes in a UK mental health organisation improved the recording of the provision of ASK, ADVISE, ACT (OFFER) and consent to referral to specialist smoking cessation support (ACT (CONSENT)).

**Methods:**

We conducted a study using a regression discontinuity design in four psychiatric hospitals with patients who received treatment from an inpatient psychosis service over 52 months (May 2012–September 2016). The system-level changes to facilitate the provision of VBA comprised: A) financially incentivising recording smoking status and offer of support (ASK and ACT (OFFER)); B) introduction of a comprehensive smoke-free policy; C) enhancements to the patient electronic healthcare record (EHCR) which included C1) a temporary form to record the financial incentivisation of ASK and ACT (OFFER) C2) amendments to how VBA was recorded in the EHCR and C3) the integration of a new electronic national referral system in the EHCR. The recording of ASK, ADVISE, ACT (OFFER/CONSENT) were extracted using a de-identified psychiatric case register.

**Results:**

There were 8976 admissions of 5434 unique individuals during the study period. Following A) financial incentive, the odds of recording ASK increased (OR: 1.56, 95%CI: 1.24–1.95). Following B) comprehensive smoke-free policy, the odds of recording ADVICE increased (OR: 3.36, 95%CI: 1.39–8.13). Following C1) temporary recording form, the odds of recording ASK (OR:1.99, 95%CI:1.59–2.48) and recording ACT (OFFER) increased (OR: 4.22, 95%CI: 2.51–7.12). Following C3) electronic referral system, the odds of recording ASK (OR:1.79, 95%CI: 1.31–2.43) and ACT (OFFER; OR: 1.09, 95%CI: 0.59–1.99) increased. There was no change in recording VBA outcomes following C2) amendments to VBA recording.

**Conclusions:**

Financial incentives and the recording of incentivised outcomes, the comprehensive smoke-free policy, and the electronic referral system, were associated with increases in recording individual VBA elements, but other changes to the EHCR were not. System-level changes may facilitate staff recording of VBA provision in mental health settings.

## Background

Smoking prevalence among people with a mental health condition is up to three times greater than the wider population and higher in psychiatric inpatient settings compared with community settings [1]. Smoking is more prevalent in people who experience psychosis compared with other mental disorders [2] and is associated with poor treatment outcomes and premature mortality [3].

Tobacco dependence treatment guidelines recommend health professionals systematically identify smokers at the first opportunity, record smoking status in clinical notes, advise to quit or the best way to quit, and offer pharmacotherapy and/or a referral for intensive behavioural support and pharmacotherapy [4–6]. These initial steps of a tobacco dependence treatment pathway are variously referred to as brief interventions, with the sequence of actions known as the 5As (ask, advise, assess, assist and arrange) recommended in United States (US) tobacco dependence treatment guidelines [4] or Very Brief Advice (VBA), recommended for United Kingdom (UK) primary and secondary health services [6]. VBA is based upon the PRIME theory of motivation [7] and was designed to be used opportunistically to trigger quit attempts [8]. Its three elements include identifying smoking status (ASK), advising on how to stop (ADVISE) and offering a referral to specialist smoking cessation support (ACT (OFFER)) [8]. This differs from the traditional approach of advising people about the harms of smoking and that they should quit [9]. A systematic review found that offering assistance to quit generated more quit attempts than giving advice to quit on medical grounds [10]. VBA does not include the assessment of motivation or readiness to quit, as this is not crucial to trigger a quit attempt [11], unlike an offer of support [10]. Opportunities to trigger quit attempts may be missed if support is only offered to those who report they are motivated to quit.

Hospitalisation presents a window of opportunity to initiate tobacco dependence treatment and the provision of VBA is an essential part of a tobacco dependence treatment pathway. The provision of VBA during a hospital admission or a community treatment episode is recommended in the English National Institute for Health and Care Excellence (NICE) guidelines [6], although historically, VBA provision and treatment among hospitalised patients has been poor [3]. Health system-level changes, including enhancements to electronic healthcare records (EHCR) [12], the use of financial incentives [13], and introducing smoke-free polices [14], have the potential to improve VBA provision. A Cochrane review of 16 studies to evaluate the use of EHCR enhancements to improve smoking cessation support reported that the documentation of tobacco status and referral to cessation counselling increased following the introduction of EHCR reminders to staff [12]. These studies were conducted in general healthcare settings (14 in primary care or general practice clinics, one in a hospital setting and one in a dental setting), mostly in the US. None of the studies were conducted in mental health settings and the authors recommended that because the findings were modest, further studies were needed to increase the evidence base for enhancements to EHCRs to encourage the provision of treatment for smoking in healthcare settings. A further study conducted in Australia by Slattery et al., evaluated a multi-component intervention across 37 acute hospitals to improve VBA and treatment and found an increase in the provision of advice and referrals for telephone support (Quitline) [15]. A study conducted in primary care settings in Canada by Papadakis et al. to evaluate a multi-component intervention, including embedding smoking status questions and prompts in electronic medical records to deliver brief advice, was found to be associated with an increase in all three components of VBA [16].

Financially incentivising healthcare clinicians or organisations to deliver VBA or individual elements (e.g. just ASK or ACT) have been associated with improvements in VBA provision [17–19]. In England, VBA has been systematised in primary and secondary care settings by financially-incentivising health care provider organisations through Quality and Outcomes Framework (QOF) targets for General Practitioners, and the Commissioning for Quality Improvement and Innovation (CQUIN) payment framework for NHS secondary acute and mental health settings [13, 14]. CQUINs were established in 2009 to encourage health service providers in England to continually improve the quality of care provided to patients. They enable commissioners of services to financially reward improvements in the quality of health and social care by linking a proportion of service providers income to the achievement of local and national quality improvement goals.

Few studies have evaluated the effect of system-level changes on the provision of VBA in mental health settings. In the US, the implementation of the 5As in six community mental health centres, which included standardisation of the delivery of the 5As in medical notes, was associated with an increase in the number of patients who self-reported past 7 day abstinence but not carbon monoxide verified abstinence at 12 month follow-up [20]. Evaluation of the American Psychiatric Association nicotine dependence guidelines demonstrated a decrease in screening for tobacco use but an increase in providing cessation counselling 10 years after the guidelines were introduced [21]. A pilot study conducted in two male mental health wards in England audited amendments to an EHCR form to record smoking status and prompt referral to a specialist smoking cessation clinic [22]. The new form doubled rates of documentation of smoking status, cessation advice and offer of NRT and or referral. An evaluation of the introduction of a comprehensive smoke-free policy in a mental health hospital in England by Huddleston et al. observed that the identification and recording of smoking status decreased post implementation, but the provision of advice and the offer of support increased, 2 months post-implementation [14]. Further work is therefore needed to evaluate whether the introduction of health system-level changes in mental health settings can improve the provision of VBA.

## Methods

We aimed to assess whether three broad health system-level changes in a mental health organisation improved the recording of the provision of ASK, ADVISE, ACT (OFFER) and consent to referral (ACT (CONSENT)). The health system-level changes to facilitate the recording of the provision of VBA included: A) financial incentivisation via a CQUIN programme to identify smoking status (ASK) and offer a referral to specialist smoking cessation support (ACT (OFFER)); B) the introduction of a comprehensive smoke-free policy; C) enhancements to the EHCR including, C1) a temporary form to record the financial incentivisation of ASK and ACT (OFFER); C2) amendments to how VBA was facilitated and recorded within an existing EHCR and C3) the integration of a new electronic national referral system (NRS) in the EHCR to refer patients to stop-smoking services.

### Study design, setting and participants

We conducted a retrospective observational pre-post study using a regression discontinuity design with data collected from hospitalised patients attending psychosis services across the South London and Maudsley NHS Foundation Trust (SLaM) UK, which provides care to approximately 1.3 million people. The organisation has four hospitals, with approximately 50 wards and 800 beds delivering a range of services. The EHCR, in place since 2005, contains records from over 270,000 patients [23] and is completed by clinical staff. Participants were patients who had received treatment from an adult inpatient Psychosis Service between 1st May 2012 and 30th September 2016, including those who had more than one admission within the study period and also had a previous admission in the 6 months before. This represented the longest continuous time period for which suitable patient data were available and covered the dates of implementation of all system-level changes of interest.

### Interventions: health system-level changes to facilitate the recording of the provision of VBA

Over the study period, several system-level changes occurred within SLaM that had the potential to improve the provision of VBA and tobacco dependence treatment.

#### A) Financially incentivising ASK and ACT (OFFER) as part of a CQUIN programme (April 2014 onwards)

SLaM introduced a CQUIN programme from April 2014 which remained throughout the study period, as part of a national strategy to reduce premature mortality of people with severe mental illness [24]. Staff were instructed to assess and record cardiometabolic parameters such as body mass index and blood pressure, as well as use of alcohol, drugs and smoking status. The organisation was required to collect evidence and report to commissioners that these parameters were screened and where clinically indicated, provided with onward referral for appropriate interventions. The CQUIN included the financially incentivised recording of the provision of identifying a patient’s smoking status (ASK) and offering a referral to specialist smoking cessation support (ACT (OFFER).

#### B) Introduction of a comprehensive smoke-free policy (October 2014 onwards)

In accordance with NICE guidelines [6], an indoor smoke-free policy, which had been in place from 2008 and prohibited smoking inside hospital buildings, was changed to a comprehensive policy in 2014. This included the prohibition of smoking in hospital buildings and grounds, and the provision of tobacco dependence treatment. The latter included the provision of VBA and offer of nicotine replacement therapy (NRT) within 30 min of admission and support from a trained tobacco dependence advisor for the duration of admission. Combination NRT or varenicline and behavioural support were also included in treatment options and e-cigarette use also permitted. The policy was supported by a staff training programme.

#### C) EHCR enhancements (from July 2014 onwards)

##### C1) Introduction of a temporary form to record smoking CQUIN goals of ASK and ACT (OFFER) (29th July 2014–1st November 2014)

A new recording form was added to the EHCR for 5 months so that staff could record ASK and ACT (OFFER) and other CQUIN activity. During this 5 month period, staff were regularly prompted to complete the form so that the organisation could capture and report data to commissioners for their CQUIN payment.

##### C2) Amendments to how VBA was facilitated and recorded in the EHCR (January 2015 onwards)

Existing questions and response options within the structured field of the EHCR pertaining to smoking behaviour were amended from January 2015 (Table 1). The identification and recording of smoking status (ASK) became mandatory (i.e. staff could not progress with completing other parts of the EHCR if smoking status was not recorded) and questions and responses regarding advising about quitting (ADVISE) and offering a referral for support (ACT (OFFER)) were changed in line with the evidence base and recommendations from NICE [6, 8, 11].

**Table 1:** Amendments to recording of VBA in the EHCR

	Pre January 2015		Post January 2015	
	Smoking questions	Response options	Smoking questions	Response options
**ASK**	Does the patient smoke tobacco	Yes / No / Uncertain / Leave blank	Does the patient currently smoke tobacco?	Yes / no (mandatory)
	Is the patient motivated to try to quit?		Question removed	
**ADVISE**	Was advice to stop given to patient?		Has the patient been advised that stopping smoking is the best thing they can do for their health and free support is available?	Yes / No / Leave blank
**ACT**^a^	Was the patient referred to a smoking cessation service?		Does the patient consent to a referral to support from a specialist adviser?	
**ACT**	Was a brief intervention offered?		Question removed	

^a^Neither version of the EHCR included a question about ‘offer’ of referral, so this was inferred from the recording of yes and no to the questions about consent for referral

##### C3) Introduction of an electronic National Referral System (NRS) integrated into the EHCR (September 2015 onwards)

The NRS was integrated and embedded into the EHCR. This replaced manually referring smokers by telephone or email to a stop-smoking service. The NRS was developed by the National Centre for Smoking Cessation and Training (NCSCT) and North 51 [25] to standardise ASK within a hospital EHCR system, and to simplify the referral process. Staff were required to tick ‘yes’ to the three structured VBA questions (Table 1) to activate the referral. Patients’ details were then automatically sent securely to the NRS and forwarded to the patient’s local community stop smoking service or a hospital tobacco dependence advisor, depending on the patient’s residence at that time. Training on the NRS was integrated into existing staff training for smoking cessation.

### Data source and measures

Data were extracted from the Clinical Record Interactive Search (CRIS) system, part of the NIHR Maudsley Mental Health Biomedical Research Centre and Dementia Unit. CRIS is a de-identified psychiatric case registry and facilitates access to an anonymised EHCR and to search against structured (e.g. age) and unstructured fields (e.g. user-defined text strings). For this study, only the structured fields were used. CRIS has been used in several studies to evaluate effects of health service changes [26, 27]. Access to CRIS is within an approved governance framework.

### Patient demographic, clinical and admission characteristics

Data on age, gender, marital, occupational status and admission characteristics (i.e. number of admissions, duration and history of being legally detained in hospital for treatment) were extracted. We were unable to extract data on all previous lifetime admissions; this was therefore restricted to the last admission occurring within the previous 6 months of the current admission. Ethnicity was extracted and categorised using the method suggested by the Office for National Statistics (ONS) [28]. Primary diagnosis defined by ICD-10 disorder type was extracted and grouped according to the following categories: F20 or schizophrenia, F21–F29 (schizotypal, delusional), F30–F39 (affective disorders) or < F20, >F40 and other [29]. Data on socioeconomic deprivation were extracted based on the first part of patients’ postcodes.

### Outcomes: recording of the provision of VBA during admission

**Fig. 1:**
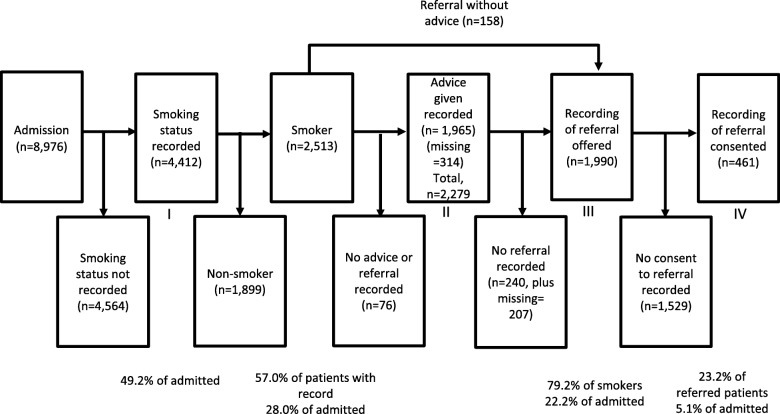
Pathway for recording of Very Brief Advice (VBA) for smoking among a sample of inpatients in a mental health setting (May 2012 to September 2016)

After admission, patients progressed through stages of the VBA pathway (Fig. 1). Variables corresponding to components of VBA (ASK, ADVISE, ACT (OFFER) and ACT (CONSENT)) were extracted from the EHCR, if provided at any point throughout the admission. ACT (OFFER) was inferred from ACT (CONSENT), as detailed in Table 1.

### Statistical analysis

After describing the sample’s demographic and clinical characteristics, we analysed the odds of patients reaching each stage of VBA, contingent on passing through the previous stage. The sample contained repeat observations for individual patients as some were admitted more than once (range: 1–23). These repeat observations were likely to be correlated (non-independent), increasing the risk of type 1 error. After using the quasi-information criterion (QIC) to determine the most appropriate correlation structure, logistic regression models for each outcome were fitted using Generalised Estimating Equations (GEE) with an exchangeable correlation structure to account for correlation of responses within individual readmitted patients [30].

We employed regression discontinuity analysis, a quasi-experimental approach which can be applied retrospectively and accounts for the longitudinal nature of the data and pre-implementation trends [31, 32]. This was achieved by segmented regression modelling pre-implementation time trend, the change in level at the time of implementation, and the change in time trend from pre-implementation to post-implementation. These were fitted using a continuous time trend variable (months since January 2011), a binary variable denoting time the health system-level change was implemented (0 = before implementation, 1 = after implementation), and an interaction term between the time trend and implementation variable respectively. The post-implementation change in time trend indicates longer-term impact. A positive change in the time trend indicates improvement over time while a negative change may signal concerns about the sustainability of the effect [33]. The effects of the temporary form to record the financial incentivisation of ASK and ACT (OFFER) which had a defined start and end date, were modelled using a binary variable for the period July to November 2014. The effect of the amendments of how VBA was facilitated and recorded was also modelled using a binary variable representing a step change from January 2015 onwards. No time interaction term was included in the model as this did not improve model fit as determined using the QIC. Effects of the implementation of the smoke-free policy and the NRS were analysed using regression discontinuity.

We adjusted for individual-level variables to account for the possible change in the characteristics of the inpatient population over time. These included age (16–24, 25–34, 35–44, 45–54, 54–64, or ≥ 65 years), sex, binary socioeconomic deprivation (most vs least deprivation) [34], lifetime history of being legally detained in hospital for treatment (yes or no), primary diagnosis (ICD-10 categories) and ethnicity (ONS categories). Adjustment was made for health and psychosocial functioning measured using the Health of the Nation Outcome Scale (HoNOS) score [35] (linear scale, range: 0–37 [36],, taken closest to the recording of a patient’s smoking status. Models adjusted for length of stay (days), in addition to a quadratic term if this was found to improve model fit. Models also adjusted for characteristics of previous admissions (last 6 months). Categories included no admission, admission with ASK not recorded, admission with ASK recorded (non-smoker), admission with ASK recorded (smoker), or admission with ACT (OFFER) recorded. Where an individual had been admitted three or more times in a six-month period, participants were coded according to the characteristics of the admission in which they had progressed furthest, from admission to referral. Number of previous lifetime admissions was not fitted, as data on admissions before November 2011 were unavailable. No attempt was made to adjust for associations between the four hospitals falling within the SLaM Trust and the outcomes investigated; patients are frequently transferred between wards/hospitals within a single admission based on case severity and bed availability.

Depending on the outcome measure, the proportion of sample observations with missing data ranged from 2.9% (ACT (OFFER)) to 15.0% (ADVISE). GEE methods are not robust to missing observations as they assume that missing observations are missing completely at random, or independent of observed or unobserved variables [30]. As this could not be assumed, GEE models were first run in Stata 15 using complete cases, and then re-fitted using imputed values obtained using multivariate imputation by chained equations using the *mi* command (20 imputations) [37–39]. Multiple imputation assumes missingness at random conditional on observed data. The number of imputations was selected based on estimates of Monte Carlo error [39].

Models were fitted within a regression discontinuity framework as described for the associations between ASK, ADVISE, ACT (OFFER) and ACT (CONSENT) and implementation of system-level changes with full adjustment for the covariates mentioned above and using both complete cases and imputed data. As only patients identified as smokers were offered advice and referrals to stop smoking services, models for these outcomes were fitted contingent on patients being smokers.

### Ethical approval

Audit approval from SLaM’s internal clinical audit department to extract data from the CRIS was obtained from their Oversight Committee. CRIS has ethical approval as an anonymised data resource for secondary analyses from Oxfordshire Research Ethics Committee.

## Results

### Descriptive analysis

Over the study period, there were 8976 admissions (including 3542 repeat admissions) (Fig. 1) of 5434 unique patients. Smoking status was identified (ASK) and recorded for 49.2% of admissions (*n* = 4412/8976), of whom 57% of patients (2513/4412) were recorded as smokers. Advice to quit (ADVISE) was recorded for 78.2% (*n* = 1965/2513) of smokers and 79.2% (1990/2513) of smokers had a recording of an offer of a referral to specialist smoking cessation support (ACT (OFFER)); 158 of the latter had a record of ACT (OFFER) without ADVISE. Consent to a referral to specialist smoking cessation support (ACT (CONSENT)) was recorded for 23.2% (*n* = 461/1990) of smokers. Of those with complete data about their smoking status and individual-level control variables, around half were males (53.7%), and 26.6% were in the 25–34 years age group; more than in other age categories (Additional file 1). Around 25% were in each of the four diagnostic groups; the mean HoNOS score was 11.8 (95% CI: 11.7–11.9) and mean length of stay 51.7 (95% CI: 49.9–53.6) days (Additional file 1).

**Fig. 2:**
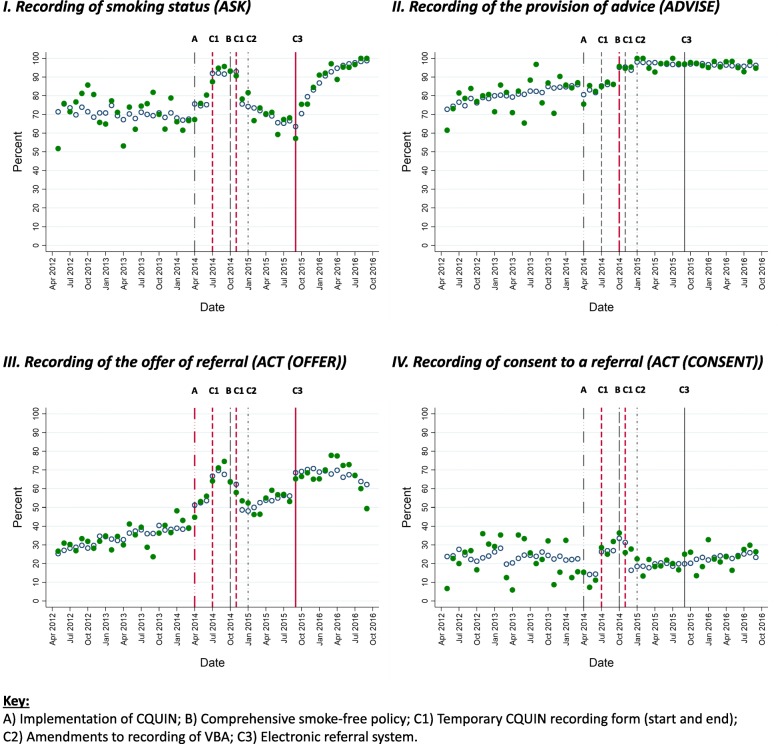
Actual (solid points) and model-estimated (hollow points) proportions of participants provided each component of VBA by month (May 2012–September 2012)

Figure 2 shows proportions of participants who reached each stage of the VBA pathway (green solid dots) and predicted proportions based on estimates from imputed models (blue hollow dots) by month. Vertical lines represent dates of health system-level changes implementation. The temporary form to record the financial incentivisation of ASK and ACT (OFFER) is represented with two lines as it was the only system-level change with defined end as well as start dates. Red lines denote system-level changes with a statistically significant immediate change in odds of a given outcome occurring or a change in the time trend (see below).

#### I. Ask

The results of the imputed model (Table 2) show that odds of recording ASK improved gradually over time as evidenced by the underling positive linear time trend (admission date variable in Table 2). Odds of recording ASK increased following the implementation of financial incentives for the CQUIN programme (A) and continued to increase while the temporary form to record smoking related CQUIN goals (C1) was in place, but not after the form was removed when the underlying time trend resumed (Fig. 2 and Table 2). Smoke-free policy implementation (B) was associated with a decrease in odds of recording ASK. Amendments to how VBA was facilitated and recorded in the EHCR (C2) were not associated with any significant change, whereas the NRS (C3) was associated with a significant increase in the odds of recording ASK. The significant post-implementation time interaction term indicates a negative trend in odds of recording ASK which counteracted the positive pre-implementation time trend before the NRS implementation.

**Table 2:** I Results of standard and imputed models for associations between system-level changes: ASK

Variable	Categories/units	Standard model^1^ (***n*** = 8380)		Imputed model (***n*** = 8976)	
		OR (95%CI)	p	OR (95%CI)	P
***CQUIN programme***					
Financial incentives	April 2014–	**1.56 (1.24–1.96)**	**< 0.001**	**1.56 (1.24–1.95)**	**< 0.001**
***Smoke-free policy***					
Smoke-free policy	Oct 2014–	**0.73 (0.57–0.93)**	**0.010**	**0.72 (0.56–0.91)**	**0.007**
Time interaction		1.02 (0.97–1.08)	0.345	1.03 (0.98–1.09)	0.210
***Enhancements to EHCR***					
C1) Temporary form	Jul 2014–Nov 2014	**1.94 (1.55–2.44)**	**< 0.001**	**1.99 (1.59–2.48)**	**< 0.001**
C2) Amendments to recording of VBA	Jan 2015–	0.97 (0.67–1.40)	0.858	0.90 (0.63–1.31)	0.593
C3) Electronic referral system (NRS)	Sept 2015–	1.36 (0.98–1.87)	0.062	**1.79 (1.31–2.43)**	**< 0.001**
Time interaction		**1.08 (1.01–1.14)**	**0.015**	**0.94 (0.89–0.99)**	**0.029**
***Admission characteristics***					
Admission date	Months (since Jan 2011)	**1.02 (1.01–1.03)**	**< 0.001**	**1.02 (1.01–1.03)**	**< 0.001**
Length of stay	Days	**1.02 (1.01–1.02)**	**< 0.001**	**1.02 (1.01–1.02)**	**< 0.001**
Length of stay squared	Days^2^	**1.00 (1.00–1.00)**	**< 0.001**	**1.00 (1.00–1.00)**	**< 0.001**
***Previous admission characteristics (last 6 months)***					
No admission		ref		ref	
Admission (smoking status not recorded)		**1.19 (1.03–1.38)**	**0.018**	**1.22 (1.06–1.40)**	**0.007**
Admission with smoking status recorded (non-smoker)		1.04 (0.81–1.35)	0.742	1.28 (1.00–1.64)	0.047
Admission with smoking status recorded (smoker and referral not offered)		**1.61 (1.06–2.45)**	**0.025**	**1.71 (1.13–2.58)**	**0.010**
Admission with referral offered		**1.28 (1.03–1.59)**	**0.028**	**1.49 (1.21–1.84)**	**< 0.001**
***Individual characteristics***					
Age	16–24	**1.34 (1.13–1.58)**	**0.001**	**1.28 (1.10–1.51)**	**0.002**
	25–34	1.11 (0.95–1.29)	0.176	1.12 (0.97–1.29)	0.116
	35–44	ref		ref	
	45–54	1.03 (0.88–1.21)	0.670	1.06 (0.91–1.23)	0.435
	54–64	1.16 (0.95–1.41)	0.143	1.14 (0.95–1.38)	0.153
	≥65	0.95 (0.66–1.37)	0.786	1.05 (0.74–1.49)	0.777
Sex	Male	ref		ref	
	Female	**0.82 (0.74–0.91)**	**< 0.001**	**0.82 (0.74–0.90)**	**< 0.001**
Ethnicity	White	ref		ref	
	African	**1.26 (1.07–1.48)**	**0.005**	**1.20 (1.03–1.40)**	**0.017**
	Caribbean	1.00 (0.83–1.22)	0.968	1.01 (0.84–1.22)	0.878
	Other black background	**1.18 (1.01–1.37)**	**0.036**	**1.20 (1.04–1.39)**	**0.011**
	Mixed	1.33 (0.97–1.83)	0.072	1.19 (0.89–1.59)	0.246
	Asian (inc. Chinese)	1.24 (0.98–1.57)	0.076	1.16 (0.93–1.45)	0.188
	Other	1.10 (0.89–1.36)	0.390	1.11 (0.91–1.35)	0.326
Socioeconomic deprivation	3 and 2 (least/medium)	ref		ref	
	1 (most deprivation)	**1.13 (1.02–1.27)**	**0.024**	1.10 (0.99–1.22)	0.065
Legally detained in hospital for treatment (lifetime)	No	ref		ref	
	Yes	**1.35 (1.18–1.53)**	**< 0.001**	**1.39 (1.22–1.57)**	**< 0.001**
HoNOS score	HoNOS scale (0–37)	1.01 (1.00–1.02)	0.073	1.01 (1.00–1.02)	0.061
Diagnosis (ICD-10 disorder type)	F30–F39 (affective disorders)	ref		ref	
	F20 or schizophrenia	0.98 (0.84–1.15)	0.836	0.99 (0.85–1.15)	0.879
	F21–F29 (schizotypal, delusional)	0.92 (0.79–1.07)	0.296	0.93 (0.80–1.08)	0.322
	<F20, >F40 and other^3^	**0.75 (0.65–0.87)**	**< 0.001**	**0.75 (0.66–0.86)**	**< 0.001**

^1^ Refers to complete cases

^2^ Quadratic term was 2 - negative (OR < 1)

^3^ Including (but not restricted to) anxiety, delusional and substance disorders Months since January 2012

Length of stay was positively associated with the odds of recording ASK, although the negative quadratic square term suggests that this relationship diminished over time (i.e. days at the beginning of admission had more effect than subsequent days later during admission) (Table 2). Patients with the following characteristics had higher odds of recording ASK: those who had been admitted within the last 6 months and did not have their smoking status recorded, or were previously recorded as a smoker and were not offered a referral, or had previously been offered a referral; male, aged 16–24 years, ‘African’ or ‘Other black’ backgrounds; or previous legal detainment in hospital for treatment. Patients with ICD-10 < F20, >F40 and other diagnosis types had lower odds of ASK than those with affective disorders. For those who had their smoking status recorded, the following had higher odds of being recorded a smoker: male; the most socioeconomically deprived; previous legal detainment in hospital for treatment; or a greater HoNOS score. Those aged over 54 years or of ‘African’, ‘Caribbean’, ‘Other black’, ‘Asian’ or ‘Other’ background had lower odds of being recorded a smoker.

#### Ii. Advise

Odds of recording ADVISE for smokers increased over time as evidenced by the positive and statistically significant time trend (Admission date, Table 3). Smoke-free policy implementation (B) was associated with a significant increase in the odds of recording ADVISE which was sustained over time; the proportion of patients recorded as receiving advice to quit reached > 95% after implementation. Other system-level changes did not have a significant association with the odds of recording ADVISE.

**Table 3:** II Results of standard and imputed models for associations between system-level changes: ADVISE

Variable	Categories/units	Standard model (***n*** = 2137)		Imputed model (***n*** = 2513)	
		OR (95%CI)	P	OR (95%CI)	p
***A) CQUIN Programme***					
Financial incentive	April 2014–	0.70 (0.39–1.25)	0.227	0.74 (0.42–1.30)	0.296
***B) Smoke-free policy***					
Smoke-free policy	Oct 2014–	**3.18 (1.33–7.58)**	**0.009**	**3.36 (1.39–8.13)**	**0.007**
Time interaction		0.91 (0.68–1.22)	0.523	0.89 (0.66–1.20)	0.453
***Enhancements to EHCR***					
C1) Temporary recording form	Jul 2014–Nov 2014	1.23 (0.69–2.22)	0.481	1.23 (0.70–2.19)	0.471
C2) Amendments to recording of VBA	Jan 2015–	3.37 (0.44–24.00)	0.225	3.62 (0.50–26.21)	0.202
C3) Electronic referral system (NRS)	Sept 2015–	1.33 (0.25–7.15)	0.744	1.34 (0.25–7.19)	0.730
Time interaction		1.03 (0.75–1.41)	0.860	1.06 (0.77–1.45)	0.716
***Admission characteristics***					
Admission date	Months (since Jan 2011)	**1.03 (1.01–1.06)**	**0.019**	**1.03 (1.00–1.06)**	**0.028**
Length of stay	Days	1.00 (1.00–1.00)	0.105	1.00 (1.00–1.00)	0.091
Length of stay squared	Days	N/A		N/A	
***Previous admission characteristics (last 6 months)***					
No admission		ref		ref	
Admission (smoking status not recorded)		1.15 (0.76–1.75)	0.502	1.08 (0.72–1.63)	0.717
Admission with smoking status recorded (non-smoker)		**0.16 (0.06–0.46)**	**< 0.001**	**0.16 (0.06–0.44)**	**< 0.001**
Admission with smoking status recorded (smoker and referral not offered)		1.51 (0.61–3.78)	0.375	1.39 (0.54–3.56)	0.495
Admission with referral offered		**1.67 (1.00–2.79)**	**0.052**	**1.68 (1.01–2.80)**	**0.047**
***Individual characteristics***					
Age	16–24	0.79 (0.48–1.29)	0.342	0.82 (0.50–1.33)	0.411
	25–34	0.77 (0.49–1.22)	0.254	0.81 (0.52–1.26)	0.356
	35–44	ref		ref	
	45–54	0.84 (0.53–1.36)	0.482	0.86 (0.54–1.36)	0.514
	54–64	0.99 (0.52–1.89)	0.983	1.08 (0.58–2.01)	0.816
	≥65	1.13 (0.32–4.01)	0.850	1.19 (0.34–4.15)	0.790
Sex	Male	ref		ref	
	Female	1.02 (0.72–1.43)	0.919	1.04 (0.74–1.46)	0.802
Ethnicity	White	ref		ref	
	African	1.18 (0.69–2.00)	0.549	1.08 (0.63–1.86)	0.774
	Caribbean	0.94 (0.54–1.63)	0.816	0.97 (0.56–1.68)	0.914
	Other black background	1.28 (0.83–1.98)	0.267	1.32 (0.87–2.01)	0.192
	Mixed	1.35 (0.57–3.24)	0.498	1.43 (0.87–2.01)	0.192
	Asian (inc. Chinese)	1.07 (0.47–2.45)	0.875	1.14 (0.46–2.78)	0.780
	Other	1.12 (0.58–2.16)	0.746	1.09 (0.56–2.11)	0.805
Socioeconomic deprivation	3 and 2 (least/medium)	ref		ref	
	1 (most deprivation)	0.87 (0.61–1.23)	0.431	0.83 (0.58–1.19)	0.316
Legally detained in hospital for treatment (lifetime)	No	ref		ref	
	Yes	1.12 (0.74–1.70)	0.586	1.13 (0.74–1.72)	0.571
HoNOS score	HoNOS scale (0–37)	1.03 (1.00–1.07)	0.092	1.03 (1.00–1.07)	0.074
Diagnosis (ICD-10 disorder type)	F30–F39 (affective disorders)	ref		ref	
	F20 or schizophrenia	1.20 (0.75–1.93)	0.447	1.19 (0.74–1.93)	0.478
	F21–F29 (schizotypal, delusional)	1.00 (0.62–1.61)	0.987	0.97 (0.61–1.56)	0.909
	<F20, >F40 and other	0.70 (0.45–1.10)	0.125	0.72 (0.46–1.14)	0.163

While none of the individual-level characteristics had a significant association with odds of recording ADVISE, odds were higher for individuals who had been offered a referral in a previous admission within the last 6 months, and lower for current smokers who were identified as non-smokers in a previous admission.

#### Iii. (Act (offer))

There was no discernible underlying time trend independent of any system-level change in the odds of recording ACT (OFFER) (assessed indirectly, see Table 1) over the period studied. There was a significant but temporary increase in odds of recording ACT (OFFER) while the temporary recording form (C1) was active (Table 4, Fig. 2). Although there was no immediate change in recording ACT (OFFER) instantly following the introduction of the NRS (C3), the positive and significant time interaction term indicates that there was a steady improvement in recording of ACT (OFFER) that occurred over a period of several months. Other health system-level changes did not have a significant association with the odds of recording ACT (OFFER).

**Table 4:** III Results of standard and imputed models for associations between system-level changes: ACT (OFFER)

Variable	Categories/units	Standard model (***n*** = 2440)		Imputed model (***n*** = 2513)	
		OR (95%CI)	P	OR (95%CI)	p
**A)*CQUIN programme***					
Financial incentive	April 2014–	1.49 (0.93–2.40)	0.099	1.53 (0.95–2.45)	0.078
**B)*Smoke-free policy***					
Smoke-free policy	Oct 2014–	1.15 (0.63–2.09)	0.650	1.17 (0.64–2.12)	0.611
Time interaction		0.98 (0.88–1.08)	0.675	0.97 (0.88–1.08)	0.605
**C)*Enhancements to EHCR***					
C1) Temporary form	Jul 2014–Nov 2014	**4.18 (2.47–7.05)**	**< 0.001**	**4.22 (2.51–7.12)**	**< 0.001**
C2) Amendments to recording of VBA	Jan 2015–	0.86 (0.39–1.90)	0.710	0.87 (0.40–1.90)	0.727
C3) Electronic referral system (NRS)	Sept 2015–	1.05 (0.57–1.95)	0.868	1.09 (0.59–1.99)	0.783
**Time interaction**		**1.42 (1.25–1.62)**	**< 0.001**	**1.43 (1.26–1.62)**	**< 0.001**
***Admission characteristics***					
Admission date	Months (since Jan 2011)	0.99 (0.96–1.01)	0.272	0.99 (0.96–1.01)	0.255
Length of stay	Days	**1.00 (1.00–1.00)**	**< 0.001**	1.00* (1.00–1.00)	**< 0.001**
Length of stay squared	Days	N/A		N/A	
***Previous admission characteristics (last 6 months)***					
No admission		ref		ref	
Admission (smoking status not recorded)		1.13 (0.84–1.54)	0.415	1.13 (0.83–1.53)	0.432
Admission with smoking status recorded (non-smoker)		1.94 (0.56–6.73)	0.295	1.91 (0.55–6.62)	0.305
Admission with smoking status recorded (smoker and referral not offered)		**0.50 (0.30–0.84)**	**0.009**	**0.50 (0.30–0.84)**	**0.009**
Admission with referral offered		**3.48 (2.22–5.45)**	**< 0.001**	**3.47 (2.22–5.43)**	**< 0.001**
***Individual characteristics***					
Age	16–24	**0.66 (0.46–0.93)**	**0.017**	**0.65 (0.46–0.92)**	**0.014**
	25–34	0.78 (0.56–1.08)	0.129	0.79 (0.57–1.09)	0.144
	35–44	ref		ref	
	45–54	0.71 (0.51–1.00)	0.050	0.72 (0.52–1.01)	0.055
	54–64	0.68 (0.44–1.05)	0.083	0.70 (0.45–1.07)	0.099
	≥65	1.20 (0.47–3.07)	0.707	1.20 (0.47–3.06)	0.710
Sex	Male	ref		ref	
	Female	1.01 (0.80–1.28)	0.933	1.03 (0.81–1.30)	0.807
Ethnicity	White	ref		ref	
	African	0.88 (0.62–1.26)	0.496	0.91 (0.64–1.29)	0.595
	Caribbean	1.14 (0.76–1.71)	0.541	1.15 (0.77–1.73)	0.496
	Other black background	0.86 (0.64–1.16)	0.337	0.88 (0.65–1.18)	0.390
	Mixed	1.28 (0.70–2.35)	0.427	1.29 (0.70–2.38)	0.407
	Asian (inc. Chinese)	1.01 (0.58–1.75)	0.973	1.04 (0.60–1.81)	0.883
	Other	1.06 (0.68–1.65)	0.802	1.09 (0.70–1.69)	0.708
Socioeconomic deprivation	3 and 2 (least/medium)	ref		ref	
	1 (most deprivation)	1.07 (0.84–1.35)	0.584	1.07 (0.85–1.35)	0.574
Legally detained in hospital for treatment (lifetime)	No	ref		ref	
	Yes	0.94 (0.69–1.28)	0.707	0.96 (0.71–1.30)	0.794
HoNOS score	HoNOS scale (0–37)	0.99 (0.97–1.02)	0.621	1.00 (0.97–1.02)	0.702
Diagnosis (ICD-10 disorder type)	F30–F39 (affective disorders)	ref		ref	
	F20 or schizophrenia	1.11 (0.80–1.53)	0.530	1.08 (0.79–1.49)	0.621
	F21–F29 (schizotypal, delusional)	1.27 (0.91–1.77)	0.166	1.25 (0.90–1.73)	0.191
	<F20, >F40 and other	0.97 (0.71–1.35)	0.873	0.99 (0.72–1.36)	0.944

Individuals who had a record of receiving an offer of a referral within the last 6 months were significantly more likely to have a record of receiving another in the current admission. Smokers aged 16–24 had lower odds of recording ACT (OFFER) as did confirmed smokers who were not recorded as being offered a referral in a previous admission.

#### Iv. (Act (consent))

Recording of ACT (CONSENT) was more likely while the temporary recording form (C1) was in place, but not after its removal (Additional file 2, Fig. 2). Other system-level changes were not associated with any significant change in odds of recording ACT (CONSENT) (Additional file 2).

The following characteristics had higher odds of recording ACT (CONSENT): longer hospital stay, or ‘Caribbean’ and ‘Other’ ethnicity, or experiencing greater levels of deprivation, or higher HONOS scores. No significant associations were found for any other patient characteristics.

A summary table of the effect of health system-level changes is shown in Table 5.

**Table 5:** Summary table of associations between interventions and recording of outcomes

		Outcomes		
**System-level Changes**	**ASK**	**ADVISE**	**ACT (OFFER)**	**ACT (CONSENT)**
A) Financial incentive (CQUIN)	Increase	No change	No change	No change
B) Comprehensive Smoke-free policy	Decrease	Increase	No change	No change
C) EHCR enhancements:				
C1) Temporary recording form	Increase	No change	Increase	Increase
C2) Amendments to VBA recording	No change	No change	No change	No change
C3) Electronic referral system (NRS)	Increase	No change	Increase	No change

## Discussion

To our knowledge, this is the first study to investigate the associations between health system-level changes in a psychiatric inpatient setting and the recording of VBA provision. The CQUIN programme, which financially incentivised the recording of smoking status (ASK) and the offer of a referral for specialist smoking cessation support (ACT (OFFER)) only positively influenced recording of ASK and not ACT (OFFER). The comprehensive smoke-free policy increased recording of ADVISE. The temporary form, introduced to record the financially incentivised CQUIN goals of ASK and ACT (OFFER), improved recording of ASK, ACT (OFFER) and ACT (CONSENT) but only when the form was in place. The NRS increased recording of ASK and ACT (OFFER). Finally, a number of patient characteristics, clinical characteristics and previous admissions were associated with VBA recording. Smoking prevalence of patients who had their smoking status recorded in our study (57%) is higher than in a previous study conducted in a UK mental health inpatient setting (39.3%) with an implemented comprehensive smoke-free policy [14].

### A) Financially incentivising ASK and ACT (OFFER) as part of a CQUIN programme

Similar to our findings, though in a different health care setting, previous studies conducted in primary care also found improvements in the recording of ASK following the introduction of financial incentives [17, 18]. However, in contrast to our findings, these studies also found an improvement in ADVISE and referrals.

### B) Comprehensive smoke-free policy

Our finding is similar to another UK study which found the recording of ASK reduced but recording of ADVISE increased following the implementation of a comprehensive smoke-free policy [14]. It is unclear why recording of ASK decreased following policy implementation. However, the proportion of patients who had their smoking status recorded in our study was higher than observed in a previous study conducted in the same organisation between 2006 and 2011, where only 11.6% of patients had a record of their smoking status in a structured field within the EHCR [34]; suggesting an improvement over time.

### *C)* EHCR enhancements

#### C1) Introduction of a temporary form to record smoking CQUIN goals & C2) Amendments to how VBA was recorded in the EHCR

An instantaneous, short-term increase in recording of ASK, ACT (OFFER) and ACT (CONSENT) occurred when the temporary form to record CQUIN goals was introduced which reverted to the pre-implementation trend after its withdrawal. The greater impact of the temporary form to record smoking CQUIN goals of ASK and ACT (OFFER) on all VBA outcomes might have been due to increased pressure on staff to prioritise completing the temporary form to ensure the organisation was remunerated during the 3 months the form was in place. The increase in the recording of ACT (CONSENT) may be attributed to the increased salience of participants’ smoking status among clinical staff which influenced attempts by staff to elicit consent.

In contrast to our findings that amendments to how VBA is facilitated and recorded in the EHCR were not associated with any outcome, previous studies in other healthcare settings found improvements in the recording of smoking status following enhancements to VBA recording in a patient’s EHCR [12]. Mandatory recording of smoking status is an international recommendation from the World Health Organisation (Framework Convention on Tobacco Control, Article 14) to develop an infrastructure to support tobacco cessation and treatment of tobacco dependence [40]. While the reason for the lack of effect of mandatory smoking status recording is unclear, there may be an element of psychological reactance by staff which undermined compliance [41].

#### C3) Electronic national referral system (NRS)

Our finding of a positive association between implementation of the NRS and recording of ASK has not been found previously whereas the improvement in odds of the recording offer of a referral (ACT (OFFER)) to smoking cessation services has [12, 42]. The improvement in recording of ACT (OFFER) following implementation of the NRS suggests that adopting a more streamlined approach for patient referral to specialist stop-smoking support may be effective in facilitating its provision.

### Relationship between smoking, VBA and patient characteristics

Certain demographic characteristics were associated with VBA recording and being identified as a smoker. Younger age (16–24 years) was associated with higher odds of recording ASK and lower odds of recording ACT (OFFER), in contrast to previous research conducted in primary care which identified that younger smokers (aged 18–24 years) were less likely to have their smoking status recorded and more likely to receive smoking cessation treatment compared with smokers aged 25–75 and over [43]. Similar to a previous study conducted in our organisation, we found that being female was associated lower odds of having their smoking status recorded [34]. Patients from African or other black ethnicities had higher odds of ASK similar to previous research [44]. Additionally, we found that patients living in areas with a greater level of deprivation were more likely to be identified as smokers and have a record of ACT (CONSENT), as corroborated by previous studies [45]. Patients with more severe mental health symptoms were more likely to be recorded as smokers and had higher odds of recording ACT (CONSENT). These findings are supported by previous research [46, 47] and contrary to clinicians’ beliefs that such patients are not interested in quitting [48].

### Implications for clinical practice and recommendations for future research

The implementation of CQUINs to financially incentivise the recording of VBA may improve staff behaviour to do this, but it appears that a concerted focus by managers to engage staff in this clinical activity is needed to have the optimal effect. The gradual improvement in recording of ACT (OFFER) following implementation of the NRS, rather than an instant step change, suggests that although this had a sustained, long-term impact, this was realised over an extended period. This may suggest that time is needed for clinical staff to become proficient with the NRS and for them to have confidence that the system is effective.

Failure to record smoking status in the EHCR resulted in the loss of 4564 (50.8%) of the 8976 participants admitted over the period studied from the VBA pathway, depriving non-identified smokers of the opportunity to receive advice and support. Meanwhile, 1529 (76.8%) of the 1990 patients offered support did not consent to a referral to specialist cessation support (Fig. 2). Although ASK is the most important intervention in terms of absolute loss of patients from the pathway and in terms of potential for interventions to improve patient throughput to smoking cessation support, any impact of improvements in ASK would be limited by the fact that less than 25% of patients offered a referral gave consent. To address this, further research is needed to evaluate the feasibility of an opt-out model in mental health settings [3] which involves automatically referring all smokers to specialist support, rather than smokers having to opt-in to treatment. This is common in maternity services and has been shown to improve referral rates for specialist support [49] and its feasibility has been evaluated in acute hospitals [50]. Since this study was conducted, a CQUIN to specifically financially-incentivise all three stages of VBA has been introduced in mental health inpatient settings in England [51], which warrants further investigation.

Furthermore, whilst 158 participants identified as smokers (6.3%) were recorded as being offered a referral to specialist support without first receiving advice, 240 of the 1965 patients who recorded as receiving advice (12.2%) were not recorded as being offered a referral. This suggests that, in the study’s setting, greater efforts could have been made to link provision of advice with offer of a referral.

### Limitations and strengths

The study is limited due to its reliance on fidelity of VBA recording; it is conceivable that VBA may have been provided without having been recorded. Additionally, our source of data collection (CRIS) does not contain information on details of the staff (e.g. job role) who provided VBA, therefore, we were unable to adjust for any difference in personnel pre and post interventions, which may have contributed to changes in the recording of the provision of VBA. Another limitation is that we did not extract data on whether the participants had a secondary mental health diagnosis and were therefore unable to test associations between secondary conditions and mental health multimorbidity, and the delivery of VBA. We also we could not obtain data about the proportion of patients who attended specialist stop-smoking services and their quit rates after providing consent for a referral.

The degree to which our results from one mental health organisation are generalisable to other settings in the UK and internationally is uncertain. The use of retrospective data restricted our choice of study design, and precluded use of a true experimental design such as a randomised controlled trial. The regression discontinuity design assumes that whether a given in-patient is admitted before or after each system-level change is a random process, and that in-patients cannot (or do not attempt to) control whether or not they are affected by a system-level change. Our interpretation of the results also assumes that potential effects of system-level changes are not ‘contaminated’ by those of other interventions occurring at or around the same time [52]. Although causal inferences as to the effects of changes in policy could potentially be drawn from our analysis, this relies on the assumption of a hard cut-off in which policies are fully implemented immediately after their intended start date and applicable to all in-patients. While patients in our sample known to SLaM services prior to October 2014 were made aware of the comprehensive smoke-free policy coming into effect and patients continued to be made aware of the policy pre-admission, it is unlikely that patients could actively choose to be admitted either before or after this system-level change (or be motivated to change their date of admission as a result of the policy). Patients were unaware of the other system-level changes occurring in the organisation. Additionally, we are unaware of any other system-level changes relevant to the outcomes we investigated occurring within the period studied.

Strengths of the study include a large dataset including routinely recorded clinical data covering 8976 admissions over a period of 52 months. To our knowledge, no other study has evaluated such system-level interventions on the recording of VBA provision in mental health settings.

## Conclusions

Health system-level changes may facilitate the recording of VBA provision in mental health inpatient settings. Financial incentives through CQUIN programmes with a particular focus on encouraging staff to engage with the CQUIN was associated with improvements. The comprehensive smoke-free policy, and the electronic NRS, were associated with increases in recording individual VBA elements, but other changes to the EHCR were not. We found an overall increase in the recording of smoking status over time [34], suggesting that staff behaviour to engage with the first step of VBA may be improving over time. Offering a referral for support to quit is helped by integrating an electronic NRS, though time is also needed for staff to become proficient in its use.

## Supplementary information

**Additional file 1.**

**Additional file 2.**

## Data Availability

The data that support the findings of this study are available from the Clinical Record Interactive Search (CRIS) system, which is part of the NIHR Maudsley Mental Health Biomedical Research Centre and Dementia Unit. However, restrictions apply to the availability of these data, which were used under license for the current study, and so are not publicly available. Access to CRIS is within an approved governance framework, therefore data are accessible if approval is sought. For further information about CRIS please contact administrator@slam.nhs.uk.

## Abbreviations

ASK: Identifying smoking status
ADVISE: Advising on how to stop
ACT (OFFER): Offering a referral to specialist smoking cessation support
ACT (CONSENT): Consent to a referral to specialist smoking cessation support
CRIS: Clinical record interactive search
CQUIN: Commissioning for quality improvement and innovation
EHCR: Electronic healthcare record
GEE: Generalised estimating equations
HoNOS: Health of the nation outcome scale
NCSCT: National centre for smoking cessation and training
NHS: National health service
NICE: National institute for health and clinical excellence
NRT: Nicotine replacement therapy
NRS: National referral system
ONS: Office for national statistics
QIC: Quasi-information criterion
SLaM: South London and Maudsley
QOF: Quality and outcomes framework
UK: United Kingdom
US: United States
VBA: Very brief advice
5As: Ask, advise, assess, assist and arrange
