# Supplementary material for: An observational study of system-level changes to improve the recording of very brief advice for smoking cessation in an inpatient mental health setting
Source: BMC Public Health. 2020 Apr 25;20:559. doi: 10.1186/s12889-020-08672-y (PMC7183585; doi:10.1186/s12889-020-08672-y)
Supplement: Supplementary file 1 — Additional file 1. [file 12889_2020_8672_MOESM1_ESM.docx]

| **Additional file 1 Table of Characteristics of the analytic samples for recording of smoking status, smoking status, recording of advice, offer of referral and consent to referral (complete cases) among inpatients (May 2012–September 2016)** | | | | | | | | | | | |
| --- | --- | --- | --- | --- | --- | --- | --- | --- | --- | --- | --- |
| **Variable** | **Categories** | **I. ASK** | | **Smoking status** | | **II. ADVICE** | | **III. ACT(OFFER)** | | **IV. ACT(CONSENT)** | |
|  |  | **N** | **%** | **n** | **%** | **n** | **%** | **n** | **%** | **n** | **%** |
| Total |  | 8,380 | 100.0 | 4,271 | 100.0 | 2,137 | 100.0 | 2,440 | 100.0 | 1,927 | 100.0 |
| Outcome (see columns) | Yes | 4,270 | 51.0 | 2,441 | 57.2 | 1,910 | 89.4 | 1,927 | 79.0 | 447 | 23.2 |
|  | No | 4,110 | 49.0 | 1,830 | 42.8 | 227 | 10.6 | 513 | 21.0 | 1480 | 76.8 |
| Age | 16–24 | 1,482 | 17.7 | 812 | 19.0 | 425 | 19.9 | 486 | 19.9 | 369 | 19.1 |
|  | 25–34 | 2,229 | 26.6 | 1,161 | 27.2 | 582 | 27.2 | 670 | 27.5 | 533 | 27.7 |
|  | 35–44 | 1,827 | 21.8 | 846 | 19.8 | 438 | 20.5 | 493 | 20.2 | 407 | 21.1 |
|  | 45–54 | 1,777 | 21.2 | 880 | 20.6 | 445 | 20.8 | 514 | 21.1 | 398 | 20.7 |
|  | 54–64 | 870 | 10.4 | 463 | 10.8 | 200 | 9.4 | 229 | 9.4 | 178 | 9.2 |
|  | ≥65 | 195 | 2.3 | 109 | 2.6 | 47 | 2.2 | 48 | 2.0 | 42 | 2.2 |
| Gender | Male | 4,501 | 53.7 | 2,439 | 57.1 | 1,428 | 66.8 | 1,634 | 67.0 | 1,288 | 66.8 |
|  | Female | 3,879 | 46.3 | 1,832 | 42.9 | 709 | 33.2 | 804 | 33.0 | 639 | 33.2 |
| Ethnicity | White | 3,603 | 43.0 | 1,640 | 38.4 | 935 | 43.8 | 1,075 | 44.1 | 850 | 44.1 |
|  | African | 1,259 | 15.0 | 714 | 16.7 | 257 | 12.0 | 297 | 12.2 | 231 | 12.0 |
|  | Caribbean | 747 | 8.9 | 393 | 9.2 | 207 | 9.7 | 225 | 9.2 | 183 | 9.5 |
|  | Other black background | 1,519 | 18.1 | 859 | 20.1 | 427 | 20.0 | 482 | 19.8 | 374 | 19.4 |
|  | Mixed | 255 | 3.0 | 145 | 3.4 | 86 | 4.0 | 95 | 3.9 | 79 | 4.1 |
|  | Asian (inc. Chinese) | 435 | 5.2 | 226 | 5.3 | 81 | 3.8 | 95 | 3.9 | 74 | 3.8 |
|  | Other | 562 | 6.7 | 294 | 6.9 | 144 | 6.7 | 171 | 7.0 | 136 | 7.1 |
| Socioeconomic deprivation | 3 and 2 (least/medium) | 5,692 | 67.9 | 2,938 | 68.8 | 1,519 | 71.1 | 1,726 | 70.7 | 1,366 | 70.9 |
|  | 1 (most deprivation) | 2,688 | 32.1 | 1,333 | 31.2 | 618 | 28.9 | 714 | 29.3 | 561 | 29.1 |
| Legally detained in hospital for treatment (lifetime) | No | 2,058 | 24.6 | 873 | 20.4 | 419 | 19.6 | 477 | 19.5 | 393 | 20.4 |
|  | Yes | 6,322 | 75.4 | 3,398 | 79.6 | 1,718 | 80.4 | 1,963 | 80.5 | 1,534 | 79.6 |
| Diagnosis (ICD-10 disorder type) | F20 or schizophrenia | 2,161 | 25.8 | 1,278 | 29.9 | 692 | 32.4 | 780 | 32.0 | 627 | 32.5 |
|  | F21–F29 (schizotypal, delusional) | 1,963 | 23.4 | 1,110 | 26.0 | 519 | 24.3 | 590 | 24.2 | 474 | 24.6 |
|  | F30–F39 (affective disorders) | 1,773 | 21.2 | 877 | 20.5 | 396 | 18.5 | 450 | 18.4 | 345 | 17.9 |
|  | <F20, >F40 and other^1^ | 2,483 | 29.6 | 1,006 | 23.6 | 530 | 24.8 | 620 | 25.4 | 481 | 25.0 |
| Previous admission characteristics (last 6 months) | No admission | 6,314 | 75.3 | 3,165 | 74.1 | 1,506 | 70.5 | 1,730 | 70.9 | 1,345 | 69.8 |
|  | Admission (smoking status not recorded) | 1,114 | 13.3 | 494 | 11.6 | 265 | 12.4 | 305 | 12.5 | 232 | 12.0 |
|  | Admission with smoking status recorded (non-smoker) | 342 | 4.1 | 218 | 5.1 | 24 | 1.1 | 29 | 1.2 | 26 | 1.3 |
|  | Admission with smoking status recorded (smoker) | 117 | 1.4 | 75 | 1.8 | 53 | 2.5 | 70 | 2.9 | 38 | 2.0 |
|  | Admission with referral offered | 493 | 5.9 | 319 | 7.5 | 289 | 13.5 | 306 | 12.5 | 286 | 14.8 |
|  | **Units** | **mean (95% CI)** | | **mean (95% CI)** | | **mean (95% CI)** | | **mean (95% CI)** | | **mean (95% CI)** | |
| HoNOS score | HoNOS scale (0–37) | 11.8 (11.7 – 11.9) | | 12.1 (11.9 – 12.2) | | 12.3 (12.1 – 12.5) | | 12.4 (12.2 – 12.5) | | 12.4 (12.2 – 12.6) | |
| Length of stay | Days | 51.7 (49.9 – 53.6) | | 72.4 (69.2 – 75.7) | | 75.7 (70.1 – 80.6) | | 74.7 (70.1 – 79.2) | | 79.6 (74.1 – 85.1) | |
| ^1^Including (but not restricted to) anxiety, delusional and substance disorders. | | | | | | | | | | | |
