# Supplementary material for: An observational study of system-level changes to improve the recording of very brief advice for smoking cessation in an inpatient mental health setting
Source: BMC Public Health. 2020 Apr 25;20:559. doi: 10.1186/s12889-020-08672-y (PMC7183585; doi:10.1186/s12889-020-08672-y)
Supplement: Supplementary file 2 — Additional file 2. [file 12889_2020_8672_MOESM2_ESM.docx]

| **Additional file 2. IV Results of standard and imputed models for associations between system-level changes: ACT(CONSENT)** | | | | | | |
| --- | --- | --- | --- | --- | --- | --- |
| **Variable** | **Categories/units** | | **Standard model (n=1,927)** | | **Imputed model (n=1,990)** | |
|  |  |  | **OR (95%CI)** | **p** | **OR (95%CI)** | **p** |
| ***A) CQUIN Programme*** | | |  |  |  |  |
| Financial incentive | | Apr 2014– | 0.65 (0.35 – 1.18) | 0.157 | 0.63 (0.34 – 1.15) | 0.130 |
| ***B) Smoke-free policy*** | | |  |  |  |  |
| Smoke-free policy | | Oct 2014– | 1.25 (0.78 – 1.98) | 0.352 | 1.28 (0.81 – 2.02) | 0.286 |
| Time interaction | |  | 1.02 (0.90 – 1.16) | 0.747 | 1.02 (0.90 – 1.16) | 0.793 |
| ***C) Enhancements to EHCR*** | | |  |  |  |  |
| C1) Temporary form | Jul 2014–Nov 2014 | | **2.20 (1.34 – 3.63)** | **0.002** | **2.27 (1.38 – 3.74)** | **0.001** |
| C2) Amendments to recording of VBA | | Jan 2015– | 1.02 (0.41 – 2.55) | 0.960 | 1.02 (0.41 – 2.53) | 0.961 |
| C3) Electronic referral system (NRS) | | Sept 2015– | 1.11 (0.53 – 2.35) | 0.775 | 1.09 (0.52 – 2.29) | 0.811 |
| Time interaction | |  | 1.00 (0.87 – 1.15) | 0.999 | 1.01 (0.88 – 1.16) | 0.870 |
| ***Admission characteristics*** | | |  |  |  |  |
| Admission date | Months (since Jan 2011) | | 0.99 (0.96 – 1.02) | 0.640 | 0.99 (0.96 – 1.02) | 0.640 |
| Length of stay | Days | | **1.00 (1.00 – 1.00)** | **<0.001** | **1.00* (1.00 – 1.00)** | **<0.001** |
| Length of stay squared | Days | | N/A |  | N/A |  |
| ***Previous admission characteristics (last 6 months)*** | | |  |  |  |  |
| No admission | | | ref |  | ref |  |
| Admission (smoking status not recorded) | | | 1.02 (0.73 – 1.43) | 0.913 | 1.01 (0.72 – 1.42) | 0.936 |
| Admission with smoking status recorded (non-smoker) | | | 0.87 (0.33 – 2.31) | 0.776 | 0.88 (0.33 – 2.35) | 0.798 |
| Admission with smoking status recorded (smoker and referral not offered) | | | 0.75 (0.33 – 1.71) | 0.499 | 0.75 (0.33 – 1.68) | 0.479 |
| Admission with referral offered | | | 1.19 (0.89 – 1.60) | 0.233 | 1.20 (0.90 – 1.61) | 0.213 |
| ***Individual characteristics*** | | |  |  |  |  |
| Age | 16–24 | | 1.05 (0.71 – 1.55) | 0.800 | 1.03 (0.70 – 1.51) | 0.878 |
|  | 25–34 | | 1.32 (0.94 – 1.84) | 0.104 | 1.31 (0.95 – 1.82) | 0.103 |
|  | 35–44 | | ref |  | ref |  |
|  | 45–54 | | 0.98 (0.68 – 1.41) | 0.903 | 1.02 (0.71 – 1.45) | 0.923 |
|  | 54–64 | | 0.92 (0.57 – 1.50) | 0.748 | 0.90 (0.55 – 1.45) | 0.655 |
|  | ≥65 | | 0.33 (0.10 – 1.12) | 0.076 | 0.34 (0.10 – 1.13) | 0.078 |
| Sex | Male | | ref |  | ref |  |
|  | Female | | 0.84 (0.64 – 1.10) | 0.197 | 0.81 (0.62 – 1.05) | 0.115 |
| Ethnicity | White | | ref |  | ref |  |
|  | African | | 1.17 (0.78 – 1.76) | 0.447 | 1.12 (0.75 – 1.68) | 0.587 |
|  | Caribbean | | **1.56 (1.02 – 2.39)** | **0.040** | **1.56 (1.02 – 2.39)** | **0.039** |
|  | Other black background | | 1.21 (0.86 – 1.69) | 0.273 | 1.20 (0.86 – 1.68) | 0.279 |
|  | Mixed | | 1.56 (0.86 – 2.84) | 0.146 | 1.53 (0.84 – 2.77) | 0.162 |
|  | Asian (inc. Chinese) | | 1.73 (0.97 – 3.09) | 0.063 | 1.69 (0.95 – 3.01) | 0.073 |
|  | Other | | **1.83 (1.17 – 2.86)** | **0.009** | **1.76 (1.13 – 2.75)** | **0.013** |
| Socioeconomic deprivation | 3 and 2 (least/medium) | | ref |  | ref |  |
|  | 1 (most deprivation) | | **1.03 (1.01 – 1.06)** | **0.009** | **1.03 (1.01 – 1.06)** | **0.010** |
| Legally detained in hospital for treatment (lifetime) | No | | ref |  | ref |  |
|  | Yes | | **1.03 (1.01 – 1.06)** | **0.009** | 0.83 (0.61 – 1.13) | 0.233 |
| HoNOS score | HoNOS scale (0–37) | | **1.03 (1.01 – 1.06)** | **0.009** | **1.03 (1.01 – 1.06)** | **0.010** |
| Diagnosis (ICD-10 disorder type) | F30–F39 (affective disorders) | | ref |  | ref |  |
|  | F20 or schizophrenia | | 0.95 (0.66 – 1.37) | 0.801 | 0.92 (0.64 – 1.32) | 0.65 |
|  | F21–F29 (schizotypal, delusional) | | 1.05 (0.73 – 1.52) | 0.791 | 1.08 (0.75 – 1.56) | 0.661 |
|  | <F20, >F40 and other | | 1.05 (0.73 – 1.50) | 0.805 | 1.07 (0.75 – 1.52) | 0.725 |
| *(OR>1) | | | | | | |
